# Disturbed Sleep Connects Symptoms of Posttraumatic Stress Disorder and Somatization: A Network Analysis Approach

**DOI:** 10.1002/jts.22619

**Published:** 2020-11-10

**Authors:** Laurence Astill Wright, Neil P. Roberts, Kali Barawi, Natalie Simon, Stanley Zammit, Eoin McElroy, Jonathan I. Bisson

**Affiliations:** ^1^ Division of Psychological Medicine and Clinical Neurosciences Cardiff University School of Medicine Cardiff United Kingdom; ^2^ Directorate of Psychology and Psychological Therapies Cardiff & Vale University Health Board Cardiff United Kingdom; ^3^ Centre for Academic Mental Health Population Health Sciences University of Bristol Bristol United Kingdom; ^4^ Department of Neuroscience Psychology and Behaviour University of Leicester Leicester United Kingdom

## Abstract

Posttraumatic stress disorder (PTSD) and physical health problems, particularly somatic symptom disorder, are highly comorbid. Studies have only examined this co‐occurrence at the disorder level rather than assessing the associations between specific symptoms. Using network analysis to identify symptoms that act as bridges between these disorders may allow for the development of interventions to specifically target this comorbidity. We examined the association between somatization and PTSD symptoms via network analysis. This included 349 trauma‐exposed individuals recruited through the National Centre for Mental Health PTSD cohort who completed the Clinician‐Administered PTSD Scale for *DSM‐5* and the Patient Health Questionnaire–15. A total of 215 (61.6%) individuals met the *DSM‐5* diagnostic criteria for PTSD. An exploratory graph analysis identified four clusters of densely connected symptoms within the overall network: PTSD, chronic pain, gastrointestinal issues, and more general somatic complaints. Sleep difficulties played a key role in bridging PTSD and somatic symptoms. Our network analysis demonstrates the distinct nature of PTSD and somatization symptoms, with this association connected by disturbed sleep.

## 簡體及繁體中文撮要由亞洲創傷心理研究學會翻譯

JOTS‐20‐0162.R3_Astill Wright_cantonese

Traditional Chinese

睡眠障礙與創傷後壓力症與身體不適症狀關聯:一種網絡分析方法

**摘要**: 創傷後壓力症(PTSD )和身體健康問題, 特別是軀體症狀障礙, 是高度共病的。研究只考察了這種疾病水平上的共存現象, 而不是評估特定症狀之間的關聯。使用網絡分析來確定作為這些疾病之間的橋樑的症狀, 可能使針對這種共病的干預措施的發展成為可能。我們通過網絡分析研究了軀體化和PTSD症狀之間的關聯。這包括通過國家精神衛生中心創傷後壓力隊列招募的349名遭受創傷的人, 他們完成了*DSM‐5*的臨床醫生管理的創傷後壓力症量表和病人健康問卷‐15 。共有215人(61.6% )符合*DSM‐5*的PTSD診斷標準。一項探索性的圖表分析在整體網絡中確定了四組緊密聯繫的症狀:創傷後壓力症、慢性疼痛、胃腸道問題和更普遍的身體不適。睡眠困難在連接創傷後壓力症和身體不適方面起了關鍵作用。我們的網絡分析顯示了創傷後壓力和身體不適的不同性質, 這種關聯與睡眠障礙有關。

Simplified Chinese

睡眠障碍与创伤后压力症与身体不适症状关联:一种网络分析方法

**摘要**: 创伤后压力症(PTSD )和身体健康问题, 特别是躯体症状障碍, 是高度共病的。研究只考察了这种疾病水平上的共存现象, 而不是评估特定症状之间的关联。使用网络分析来确定作为这些疾病之间的桥梁的症状, 可能使针对这种共病的干预措施的发展成为可能。我们通过网络分析研究了躯体化和PTSD症状之间的关联。这包括通过国家精神卫生中心创伤后压力队列招募的349名遭受创伤的人, 他们完成了*DSM‐5*的临床医生管理的创伤后压力症量表和病人健康问卷‐15 。共有215人(61.6% )符合*DSM‐5*的PTSD诊断标准。一项探索性的图表分析在整体网络中确定了四组紧密联系的症状:创伤后压力症、慢性疼痛、胃肠道问题和更普遍的身体不适。睡眠困难在连接创伤后压力症和身体不适方面起了关键作用。我们的网络分析显示了创伤后压力和身体不适的不同性质, 这种关联与睡眠障碍有关。

Posttraumatic stress disorder (PTSD) is a common and well‐recognized psychiatric disorder that occurs following a traumatic event. Lifetime prevalence differs according to trauma type and varies between 1.3% and 8.8% (Atwoli et al., 2015). The estimated mean conditional risk of developing PTSD after any traumatic event is 4.0%, but this risk is much higher for interpersonal trauma, such as sexual assault and armed combat (Kessler et al., 2017). The disorder is characterized by symptoms of reexperiencing, avoidance, negative changes in thoughts and mood, and hypervigilance (American Psychiatric Association, [APA], 2013). Posttraumatic stress disorder is a debilitating condition that causes considerable distress and impairment in social, occupational, and interpersonal functioning, in addition to significant societal costs (Ferry et al., 2015) and a wide range of psychiatric and physical health problems (Shalev et al., 2019).

Common comorbidities with PTSD include substance abuse, suicide, depression (Bisson et al., 2015), life‐limiting infections (Song et al., 2019), coronary heart disease (Edmondson et al., 2013), Type 2 diabetes, and autoimmune disease (Scherrer et al., 2019), as well as chronic physical symptoms. Individuals with PTSD report poorer physical health‐related quality of life and more gastrointestinal and cardiac problems, musculoskeletal pain, and general health complaints compared to those without PTSD (Pacella et al., 2013). Between 50% and 80% of individuals with PTSD have chronic physical symptoms (i.e., long‐lasting abnormal bodily sensations; McAndrew et al., 2019), and 9.7% of individuals with chronic physical symptoms have PTSD, with a particularly high prevalence among those with chronic pain (20.5%; Siqveland et al., 2017). One meta‐analysis of associations between PTSD and physical symptoms found an effect size of 0.46 (κ = 16; Pacella et al., 2013). The comorbidity of PTSD and physical symptoms results in poorer prognoses, higher levels of disability, more severe symptoms, lower treatment engagement, and more opioid use (McAndrew et al., 2019). The combination of high prevalence and poorer outcomes suggests common etiological mechanisms.

Physicians have traditionally clustered physical symptoms that cannot be fully medically explained into conditions such as chronic pain syndrome, irritable bowel syndrome (IBS), chronic fatigue syndrome, tinnitus, temporomandibular joint pain, whiplash, headaches, and fibromyalgia (Afari et al., 2014). Current thinking suggests a significant influence of psychological and social factors whereby there is no clear organic cause for such distressing somatic complaints (Morina et al., 2018). The fifth edition of the *Diagnostic and Statistical Manual of Mental Disorders* (*DSM‐5*; APA, 2013) conceptualizes medically unexplained symptoms (MUS) as “somatic symptom disorders” (SSD) and requires the presence of distressing physical health complaints in association with excessive concern or preoccupation with somatic symptoms (Afari et al., 2014). This suggests a large psychobehavioral overlay in a condition with uncertain physical pathophysiology (Henningsen, 2018), with considerable personal and societal costs (Konnopka et al., 2013). Previous conceptualizations of MUS were criticized as reductive, with an ongoing debate over classification as a singular syndrome or as separate conditions, thus prompting redefinition within the *DSM‐5* (Okur Guney et al., 2019). The etiology of MUS is unclear, but there appears to be a complex interplay of biological, psychological, and social factors that cause and maintain bodily distress.

Often, SSDs involve inflammatory or immune‐related processes, and it is likely that PTSD affects global immune functioning through shifts in neurobiology in the hypothalamic–pituitary–adrenal (HPA) axis (Song et al., 2019), although we did not assess this in the current study. Furthermore, cognitive and behavioral features of PTSD, such as insomnia, depression, substance misuse, anxiety sensitivity, avoidant coping, and negative posttraumatic cognitions may, directly and indirectly, exacerbate somatic symptoms through systemic changes in inflammatory and immune functioning (Kendall‐Tackett, 2009). Negative posttraumatic cognitions, such as an individual's negative perceptions of themselves and the world, are closely related to low levels of social support and interpersonal problems and contribute to the onset and maintenance of PTSD (Alliger‐Horn et al., 2017). These negative cognitions have also been associated with chronic pain–related impairment (Porter et al., 2013).

The association between PTSD severity and somatic symptom severity may be bidirectional as they may mutually maintain one another (McAndrew et al., 2019) via both psychosocial and biological factors, with PTSD and somatic symptom comorbidity resulting in a poorer prognosis, more disability, and poorer treatment outcomes (Sharp & Harvey, 2001). This relation could also be due to confounding (e.g., related to the severity of trauma exposure). Both conditions may be independently associated with trauma exposure, with neither expressing a causal effect on the other.

To date, researchers have only examined this co‐occurrence at a disorder level by investigating either an individual syndrome or the broader category of SSD. Previous work has not provided clear evidence of any specific overlapping symptoms, which, considering the high comorbid prevalence and common underlying etiologies, requires investigation. Network analysis posits that psychiatric disorders result from the complex causal interplay between symptoms rather than as a result of a distinct underlying disease entity. We wished to use this network analysis approach by assessing symptom relatedness to examine whether there were distinct clusters of PTSD symptoms separate from physical health symptoms or if there were symptoms that acted as bridges or boundaries between the disorders.

Network analysis is an exploratory technique that allows researchers to investigate the co‐occurrence of these disorders by examining the direct associations between PTSD and physical health symptoms. Thus far, network analysis has been used in traumatic stress research to investigate the associations between PTSD, anxiety, and depression (Frewen et al., 2013); PTSD and depression (Afzali et al., 2017); and *International Classification of Diseases* (11th rev.; *ICD‐11*) PTSD and complex PTSD (CPTSD) symptom clusters (Knefel et al., 2019; McElroy et al., 2019). These findings have identified bridge symptoms (i.e., symptoms that are important in connecting densely connected clusters of nodes nested within broader networks), which may provide insight into potential mechanisms that give rise to and/or maintain comorbidity and suggest useful targets for early interventions regarding the development of comorbidity (Afzali et al., 2017).

The aims of the present research were to examine the network structure of somatization and PTSD symptoms and to assess where and to what degree these symptom domains are related. This was the first study, to the best of our knowledge, to apply network analysis to comorbid PTSD and physical health problems or to somatization more broadly. Although we expected some clustering of PTSD symptoms, with separate clustering of somatic items based on affected organ systems, the study remained exploratory with regard to which symptoms would act as bridges between PTSD and somatization.

## Method

### Participants and Procedure

Participants in this cross‐sectional network analysis were recruited via the National Centre for Mental Health (NCMH), a government‐funded Welsh research center (National Health System [NHS] Health Research Authority, 2016). The cohort was a trauma‐exposed sample of individuals who self‐reported a current or historical PTSD diagnosis or experienced a traumatic event that satisfied *DSM‐IV* Criterion A (Brewin et al., 2002). All participants in the present sample subsequently completed the Clinician‐Administered PTSD Scale for *DSM‐5* (CAPS‐5), with most screening positive for current PTSD. Individuals who were 16 years of age or older were recruited through primary and secondary mental health services as well as through online advertising strategies and a variety of social media outlets. Due to the risk of exacerbating psychological distress, we excluded individuals who had recently been a mental health inpatient or were in frequent contact with a crisis‐related intensive home treatment team. We also excluded individuals who were unable to read and write in English. In total, 196 participants who were contacted refused to participate or did not attend the initial interview. Of the 355 participants who completed the initial interview, 349 completed the CAPS‐5, forming our sample. The present study was granted ethical approval by the Wales Research Ethics Committee 2.

### Measures

#### PTSD Symptoms

We used the CAPS‐5 (CAPS‐5; Weathers et al., 2018) to assess PTSD symptoms. The CAPS‐5 is a clinician‐administered scale and is widely referred to as the gold standard in PTSD assessment (Weathers et al., 2018). The scale measures symptom severity and intensity, with responses given on a 5‐point Likert scale ranging from 0 (*absent*) to 4 (*extreme/incapacitating*). Reexperiencing symptoms (i.e., Criterion B) are measured as a sum of the intensity scores for five items. Avoidance symptoms (i.e., Criterion C) is measured as the sum of the intensity scores for two items. Negative alterations in cognitions and mood (i.e., Criterion D) and alterations in arousal and reactivity (i.e., Criterion E) are measured as the sum of seven items and six items, respectively. A *DSM‐5* PTSD diagnosis requires a minimum of one reexperiencing symptom, one avoidance symptom, and at least two symptoms relating to negative alterations in cognition and mood and two symptoms related to hyperarousal. In addition, the symptoms must have lasted for at least 1 month and must be causing significant distress or functional impairment. The CAPS‐5 has demonstrated high internal consistency (Cronbach's α = .88) and strong test–retest reliability (к = .83; Weathers et al., 2018).

#### Somatic Symptoms

We used the Patient Health Questionnaire–15 (PHQ‐15; Kroenke et al., 2002) to measure self‐reported somatic symptom severity over the past 7 days. Each of the fifteen items on the PHQ‐15 is rated on a 3‐point Likert scale ranging from 0 (*not bothered at all*) to 2 (*bothered a lot*). The total score is continuous and can range from 0 to 30; the measure can also provide a categorical measure of somatic symptom severity. A total score of 0 to 4 indicates minimal somatic symptom severity, 5 to 9 indicates low severity, 10 to 14 indicates medium severity, and 15 to 30 indicates a high level of symptom severity (Kroenke et al., 2002). The PHQ‐15 is well validated and has a sensitivity of 78% and a specificity of 71% for a *DSM‐IV* diagnosis of somatoform disorder in a primary care setting when using a cutoff score of 3 or more severe somatic symptoms over the preceding 4 weeks (van Ravesteijn et al., 2009). We removed an item related to menstruation due to a large proportion of male participants in our sample and missing data from female participants.

### Data Analysis

**Figure 1:**
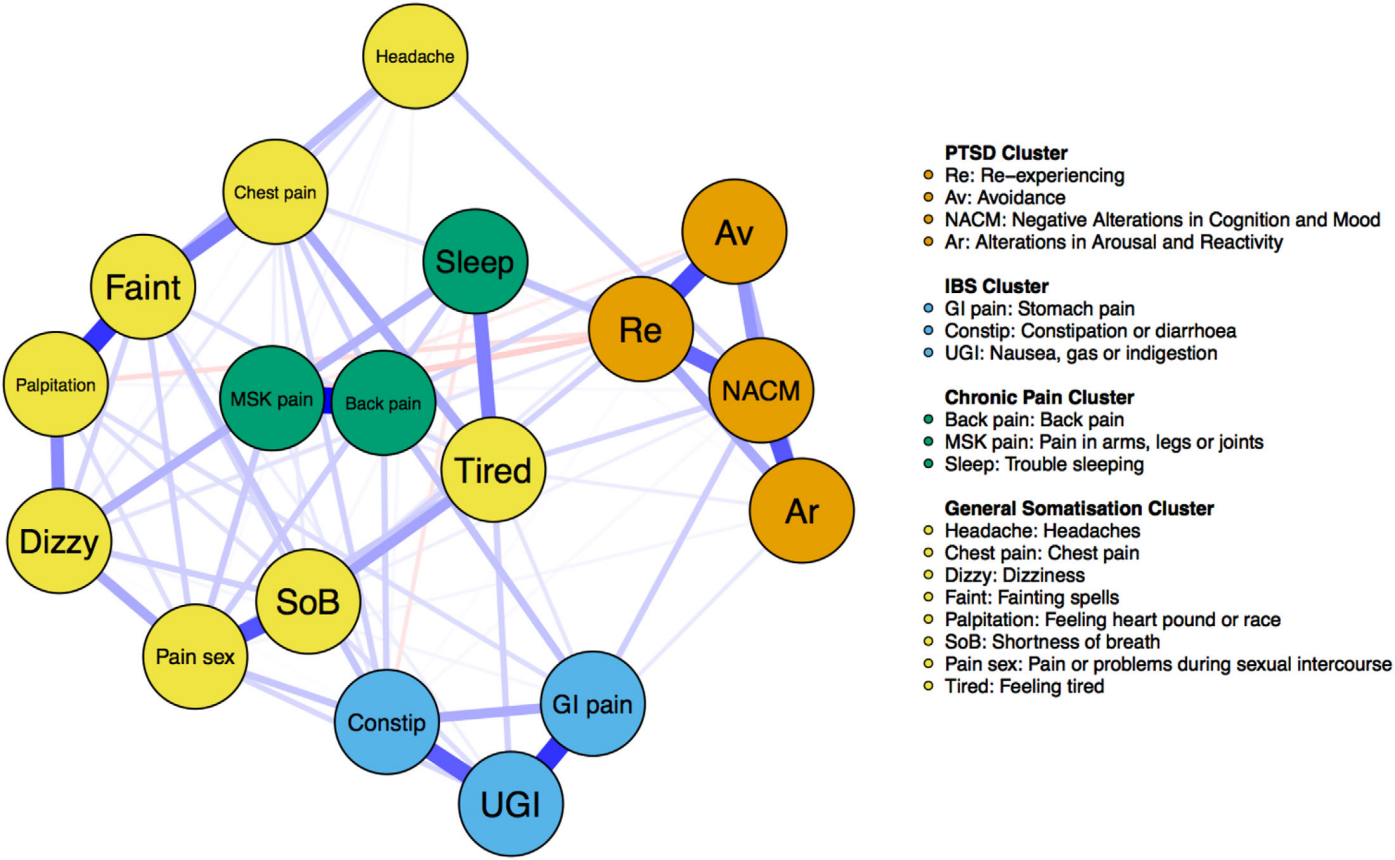
Network Depiction of Associations Among Posttraumatic Stress Disorder (PTSD) Symptoms and Symptoms Related to Physical Health *Note*. Symptoms of PTSD assessed using the Clinician‐Administered PTSD Scale for *DSM‐5*; physical health symptoms assessed using the Patient Health Questionnaire–15. Blue lines represent positive associations, with the thickness of the line indicating association strength. The color of the node represents which of the four clusters the symptom belongs to as estimated by exploratory graph analysis (testing for modularity). IBS = irritable bowel syndrome; GI = gastrointestinal; UGI = upper GI; MSK = musculoskeletal.

We used the R package “qgraph” (Epskamp et al., 2012) to estimate a network using the CAPS‐5 and PHQ‐15. To avoid topological overlap, which may upwardly bias the importance of symptoms within a network (Fried & Cramer, 2017), we created item parcels by summing items reflecting Criterion B (reexperiencing), Criterion C (avoidance), Criterion D (negative alterations in cognition and mood), and Criterion E (alterations in arousal and reactivity) using the CAPS‐5 scores. In addition, we included 14 of the PHQ‐15 items (excluding Item 4, as noted, which was only applicable to women), each of which represented a unique somatic symptom. Symptoms in the network are represented as nodes, and the connecting lines between the nodes, known as edges, represent the associations between symptoms. The appropriate correlation matrix was computed using the cor_auto function in qgraph, and this was used to estimate a regularized partial correlation network. To avoid problems associated with multiple testing, we used the graphical LASSO method to shrink edges and set very small edges to 0 using the default tuning parameter of 0.5 (Epskamp et al., 2018). This method produces a sparse network that balances fit with explanatory power (Epskamp et al., 2018). Edges within such networks can be interpreted as partial correlations, controlling for all other variables in the network. Thicker edges represent stronger associations between nodes. The Fruchterman–Reingold graphical algorithm arranges nodes based on how strongly they are associated with one another, with more weakly correlated nodes placed peripherally in the network (Epskamp et al., 2012). We tested for modularity using the “walktrap” algorithm in the Exploratory Graph Analysis (EGA) R package (Golino & Epskamp, 2017). This function detects clusters of densely connected nodes that are nested within the broader network (Figure 1).

To estimate each symptom's importance within the network, we examined node centrality (Opsahl et al., 2010). Centrality refers to the connectivity of symptoms within a network (i.e., the number and strength of connections a node has with other nodes) and is theoretically proposed to denote the importance and clinical relevance of symptoms within a given network. We used strength (i.e., the absolute sum of edge weights) as a measure of node centrality. To determine which nodes were influential in bridging symptom clusters—if clusters were identified using the walktrap algorithm, discussed earlier—we calculated bridge strength. This bridging metric was calculated in a similar way to standard centrality measures; however, this metric focuses solely on connections between clusters. Bridge centrality indices were calculated using the R package “networktools” (Jones et al., 2019). All centrality and bridge centrality indices were presented as standardized z scores, with higher scores reflecting a higher degree of importance within a network.

We used the R package “bootnet” to assess the stability of individual networks (Epskamp et al., 2018). Bootstrapping 95% confidence intervals around the network edge weights allows for the estimation of a correlation stability coefficient for centrality metrics, with values above 0.5 implying strong stability; edge‐weight differences; and centrality differences (Epskamp et al., 2018). We estimated the network using a pairwise present approach to missing data. To assess whether the missing data could affect the estimation of the network, we imputed missing PHQ‐15 values using the R package “missforest” (Supplementary Figure 4).

## Results

Of the 349 individuals who completed the CAPS‐5, 215 (61.6%) met the *DSM‐5* diagnostic criteria for PTSD, whereas 134 (38.4%) did not meet the criteria. The demographic characteristics of the sample are presented in Supplementary Table 1. Of the 349 CAPS‐5 completers, 221 completed all PHQ‐15 items, excluding the menstruation item, with 249–282 individuals completing each item. Regarding somatic symptom severity, 116 participants (52.5%) scored in the high range, 52 (23.5%) scored in the medium range, 30 (13.6%) scored in the low range, and 16 (7.2%) had a minimal level of symptom severity. As explored in Supplementary Table 2, individuals with PTSD had higher levels of somatic symptom severity than those without PTSD.

### Network Structure

We assessed the stability of the network and the validity of our findings by estimating the confidence intervals of edge weights (Fried et al., 2018). This uses a bootstrapping method that estimates the robustness of the strength centrality measure. As shown in Supplementary Figure 1, the confidence intervals were moderately wide, indicating some imprecision of the network estimation. The correlation stability coefficient for the strength centrality metric was .361, which is above the cutoff score of .25 but below the recommended score of .50 as a reliable indicator of stability (Epskamp et al., 2018)). Symptom overlap was assessed using the Goldbricker function in the networktools R package. Fewer than 25% of the correlations were significantly different for the following pairs of nodes: constipation or diarrhea and stomach pain, avoidance and reexperiencing, feeling one's heart pound or race and fainting spells, pain or problems during sexual intercourse and shortness of breath, and nausea, gas or indigestion and constipation or diarrhea. We considered the correlations between these nodes to represent meaningful connections with one another rather than redundancy. As items on both the PHQ‐15 and the CAPS‐5 (i.e., Items B2 and E6) assess disturbed sleep, we removed the CAPS‐5 items from the network. Both the pairwise present approach (Figure 1) and the imputation (Supplementary Figure 1) of missing data produced very similar networks.

### EGA Clustering

The EGA revealed a four‐cluster solution. The first cluster comprised PTSD symptoms, the second comprised chronic pain symptoms as well as poor sleep, the third comprised gastrointestinal symptoms, and the fourth was made up of more general somatic complaints (Figure 1).

### Centrality Analysis and Stability

**Figure 2:**
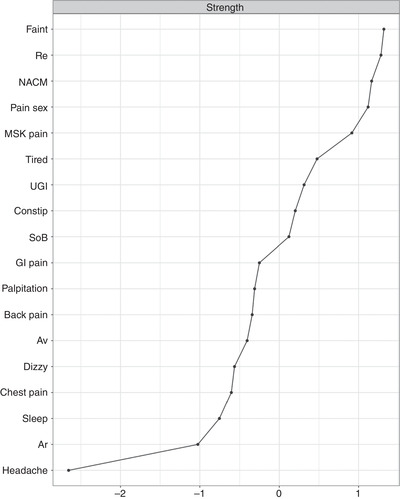
Centrality Analysis Using the Strength of Each Node *Note*. Re = reexperiencing; NACM = negative alterations in cognition and mood; MSK = musculoskeletal; GI = gastrointestinal; UGI = upper gastrointestinal; SoB = shortness of breath; AV = avoidance; AR = arousal.

**Figure 3:**
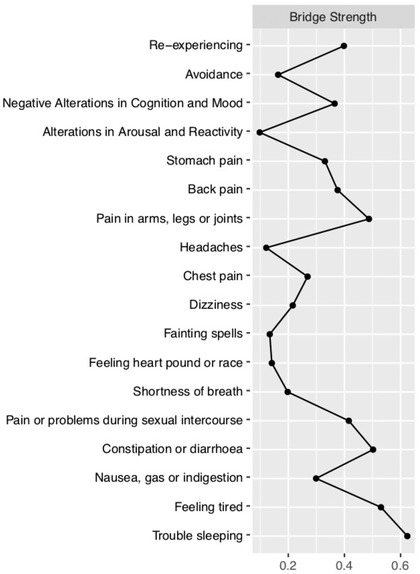
Bridge Centrality Strength Network Statistics

Centrality and bridge centrality measures are presented in Figure 2 and Figure 3. Our bootstrapping procedure supported the robustness of our centrality measures, graph structure, and edge weights (Supplementary Figures 1, 2, and 3). Fainting spells, pain or problems during sexual intercourse, and negative alterations in cognition and mood were the symptoms with the highest strength centrality. Trouble sleeping and feeling tired or having low energy were the symptoms highest in most measures of bridge centrality. The high bridge centrality of sleep difficulties and tiredness may be derived from the close associations between these two symptoms and this may drive the increased bridge centrality of the “trouble sleeping” node.

## Discussion

Through graphical LASSO networks, we were able to identify distinct traumatic stress and somatization symptom structures. The network suggests that PTSD and somatic symptoms form distinct clusters and that sleep difficulty may act as a key bridge between these domains. Sleep difficulty could be a fundamental symptom that exacerbates and maintains PTSD and somatization comorbidity, but it could also be a consequence of this comorbidity, thus resulting in a positive correlation. As sleep was assessed by both of the scales used in the present study, we removed sleep‐related CAPS‐5 items to avoid exaggerating the perceived importance of sleep within the network via overlapping measurements. It should be noted, however, that sleep disturbance is not a feature of *DSM‐5* SSD (APA 2013).

Sleep disturbance is a core symptom of PTSD, with the CAPS‐5 containing items relating to recurrent distressing dreams and difficulty falling and staying asleep (APA, 2013), which are contained within the reexperiencing and arousal nodes, respectively. Between 70% and 91% of individuals with PTSD report trouble falling or staying asleep, with between 19% and 71% experiencing nightmares (Maher et al., 2006). Sleep problems are associated with the onset, maintenance, and worsening of PTSD (Maher et al., 2006). Moreover, 64.9% of individuals who report somatic complaints also report trouble sleeping (Hanel et al., 2009), with sleep difficulties accurately predicting the onset and exacerbation of chronic pain (Finan et al., 2014). Sleep is broadly associated with poorer psychiatric outcomes (Krystal, 2012), and thus may indicate global PTSD and/or somatization severity, with more severe symptoms of either PTSD or somatization producing more severe symptoms of the other in addition to poorer treatment outcomes (Akerblom et al., 2017).

There may be causal mechanisms that relate poor sleep to PTSD and somatization, but uncertainty remains regarding the direction of causality. Mechanisms by which sleep has a causal effect on PTSD and somatization include both physical factors, such as inflammation, and psychological factors, such as cognition. Research has shown that posttraumatic cognitions may directly, via increasing negative preoccupation and perception of the somatic symptoms, and indirectly, through effects on depressive symptomatology and chronic inflammation, exacerbate somatic symptoms (Kendall‐Tackett, 2009), and sleep may have a key role in this by deleteriously affecting cognition and worsening catastrophic appraisal of somatic symptoms (Krystal, 2012). Sleep disturbance is also associated with higher levels of systematic inflammation, particularly c‐reactive protein and interleukin‐6 (Irwin et al., 2016). Some MUS, such as fibromyalgia, are well known (Littlejohn & Guymer 2018) to involve immune‐related processes, while individuals with PTSD exhibit significantly elevated levels of proinflammatory markers, suggestive of HPA axis dysfunction (Hori & Kim, 2019). Poor sleep may exacerbate systemic immune dysregulation, resulting in increased severity with regard to symptoms of somatization and PTSD.

The causality of the association between PTSD and somatization is unlikely to be unidirectional, despite the findings from one study that demonstrated little evidence that the risk of new PTSD is increased in individuals with preexisting somatization (Andreski et al., 1998). It seems more likely that the association is one of bidirectional mutual maintenance (McAndrew et al., 2019), involving both psychosocial and biological factors, which could be mediated by sleep. Posttraumatic cognitions and inflammation may have a key role in this bidirectional model whereby PTSD may cause worse somatization symptoms through poor sleep and, conversely, somatization may cause more severe PTSD, again via poor sleep.

Our EGA clustered sleep with back pain and pain in the arms, legs, or joints. Individuals with comorbid chronic pain and PTSD report more severe pain and pain‐related impairment and are more likely to have a high risk of opioid use (Morasco et al., 2013) compared to those with chronic pain alone. Between 50% and 88% of individuals with chronic pain also report insomnia (Wei et al., 2018), whereas 20% report PTSD—a higher proportion than other somatization syndromes (Siqveland et al., 2017). Thus, it is not surprising that disturbed sleep was considered a core symptom in the present network's chronic pain cluster, but sleep may also mediate the strong associations between PTSD and chronic pain that have been reported (Siqveland et al., 2017); our analyses support this strong association. Another explanation may be that sleep plays a key mediating role between chronic pain and PTSD, with chronic pain causing more severe sleep disturbances and thus exacerbating PTSD. Our analyses of somatization in adults supports and builds on previous research highlighting associations between chronic pain and sleep disturbance in young individuals (Noel et al., 2018; Pavlova et al., 2020).

The PTSD symptoms of avoidance and hyperarousal had a neutral or negative strength within the network. This indicates their lack of associations with somatization symptoms. Negative alterations in cognition and mood and reexperiencing symptoms, however, demonstrated positive connectivity within the network.

Judith Herman (1992) previously proposed somatization as one of six criteria for a diagnosis of “disorders of extreme stress not otherwise specified” (DESNOS), and although the association between physical health complaints and PTSD remains strong, these findings suggest somatization is not an integral part of the PTSD diagnosis itself. Our results support the recent conceptualization of the association between PTSD and somatization illustrated in the *DSM‐5* PTSD criteria (APA, 2013), with somatization and physical health problems remaining factors that are associated with PTSD but not included as core PTSD symptoms.

Our results support previous validation work regarding the PHQ‐15 (Leonhart et al., 2018), with a confirmatory factor analysis identifying a bifactor model that included one general symptom group and four independent organ‐specific symptom groups representing gastrointestinal symptoms, fatigue, cardiopulmonary symptoms, and pain‐related symptoms (Witthoft et al., 2016). We identified a general physical health cluster, with separate clusters for gastrointestinal symptoms and chronic pain, and although the EGA analysis did not identify a specific cardiopulmonary cluster, the three cardiac symptoms were strongly associated with one another in the network. The subjective phenomena of pain can be qualitatively different across disorders and individuals; thus, stomach pain, chest pain, and back pain exist within different symptom clusters. These findings question the unification of psychosomatic problems within SSD in the *DSM‐5* (APA, 2013), as, within the present sample, we were not able to identify discrete syndromes, although the PHQ‐15 does not measure illness‐related thoughts, feelings, and behaviors, which are a key component of the SSD diagnosis (Henningsen, 2018).

In the present study, we found that individuals with PTSD reported higher levels of somatic symptom severity than those without PTSD, and this was likely due to both biological and psychosocial factors. Physical health problems can be caused by traumatic events (Gillock et al., 2005), and traumatic stress reactions themselves can cause long‐term shifts in systemic immune functioning, with prolonged somatic health problems a result of ongoing hyperarousal (Sijbrandij et al., 2015). Unhealthy and dysfunctional coping strategies, such as tobacco, alcohol, and drug abuse, can lead to even poorer physical health (Berenz & Coffey, 2012). Seeking medical expertise via a physical health complaint may be more acceptable for some individuals who are reluctant to disclose their psychiatric symptoms due to stigma or because of the characteristic avoidance of PTSD. It is also possible that individuals with PTSD may be especially preoccupied with their somatic complaints, and hyperarousal and anxiety may lead to a catastrophic interpretation of the symptoms or an attentional bias toward negative symptoms (Bryant et al., 1999).

It is important to acknowledge some limitations to the present study that should be taken into account when interpreting the results. First, although the sample was composed of clinical cases, it was modest in size (i.e., *N* = 349), and the confidence intervals of edge weights were imprecise. Furthermore, the potential for selection bias (e.g., Berkson's bias; de Ron et al., 2019) when using clinical samples highlights the need for some caution in the interpretation of our networks. Due to sample size limitations, we were unable to assess individual PTSD symptoms as separate nodes within the network, and grouping PTSD symptoms into *DSM‐5* PTSD criteria may have created an artificial clustering of these symptoms within the network. Further replication using larger sample sizes is required to confirm our results. The cross‐sectional nature of our study prevents the determination of causality from the investigated associations. Third, our sample lacked ethnic heterogeneity, with 94% of the sample reporting their race as White. The results of this network analysis may be specific to our sample population, which includes a specific cultural context of distress following a traumatic event (Gilmoor et al., 2019); thus, it is important to confirm these findings in different cultural groups with larger samples. Our work highlights key avenues for future research, such as the association of inflammatory biomarkers in the relation between PTSD and somatization and examinations of causal effects through intensive longitudinal assessments (Gelkopf et al., 2018).

The results of our network analysis demonstrate the distinct nature of PTSD and somatization, with sleep emerging as a central factor in bridging these disorders. The present results support the validity of the PHQ‐15 as a means to differentiate between organ‐system–related syndromes and the categorization of somatization symptoms into these syndromes. Previous studies have only examined the broad latent constructs within the framework of comorbid PTSD and somatization. This was the first study of which we are aware to investigate this association on an item level. Our analyses offer key insights into the underlying mechanisms of this clinically important comorbidity, paving the way for future research aimed at investigating the role sleep plays in this association.

## Open Practices Statement

The study reported in this article was not formally preregistered. Neither the data nor the materials have been made available on a permanent third‐party archive; requests for the data or materials should be sent via email to the lead author at laurencewright@doctors.org.uk.

## Supporting information

Supporting Material

Supporting Material

Supporting Material
