# Supplementary material for: Disturbed Sleep Connects Symptoms of Posttraumatic Stress Disorder and Somatization: A Network Analysis Approach
Source: J Trauma Stress. 2020 Nov 10;34(2):375–83. doi: 10.1002/jts.22619 (PMC9943267; doi:10.1002/jts.22619)

Supplementary Figure 1: Edge weights 95% confidence intervals using bootstrapping procedure
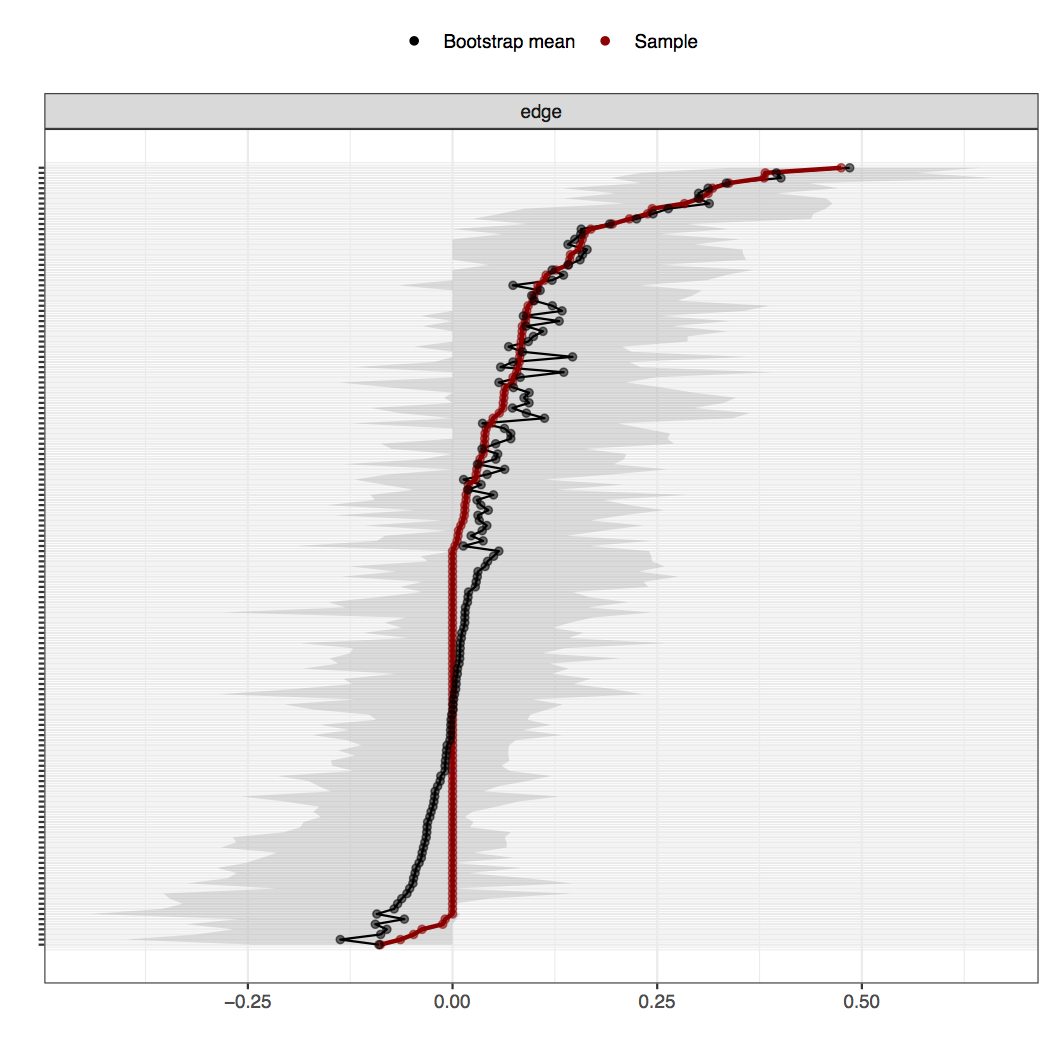


Supplementary Figure 2: Bootstrapped difference test of edge weights. Black boxes indicate significant differences in edge weights, gray boxes indicate no significant differences and standardised edge weights are documented in the diagonal.


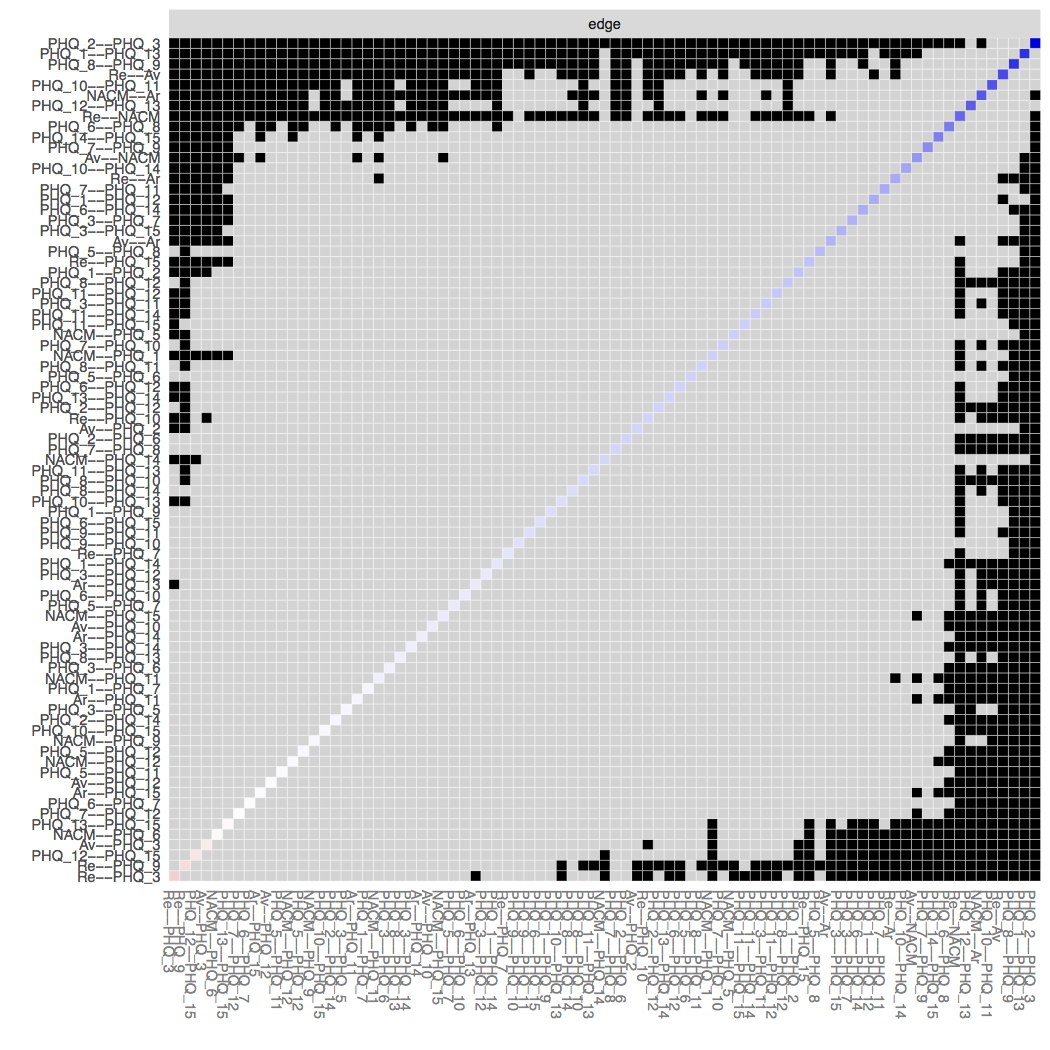


Supplementary Figure 3: Centrality stability plot showing the average correlations between centrality index of the original network overlaid with bootstrapped samples data.
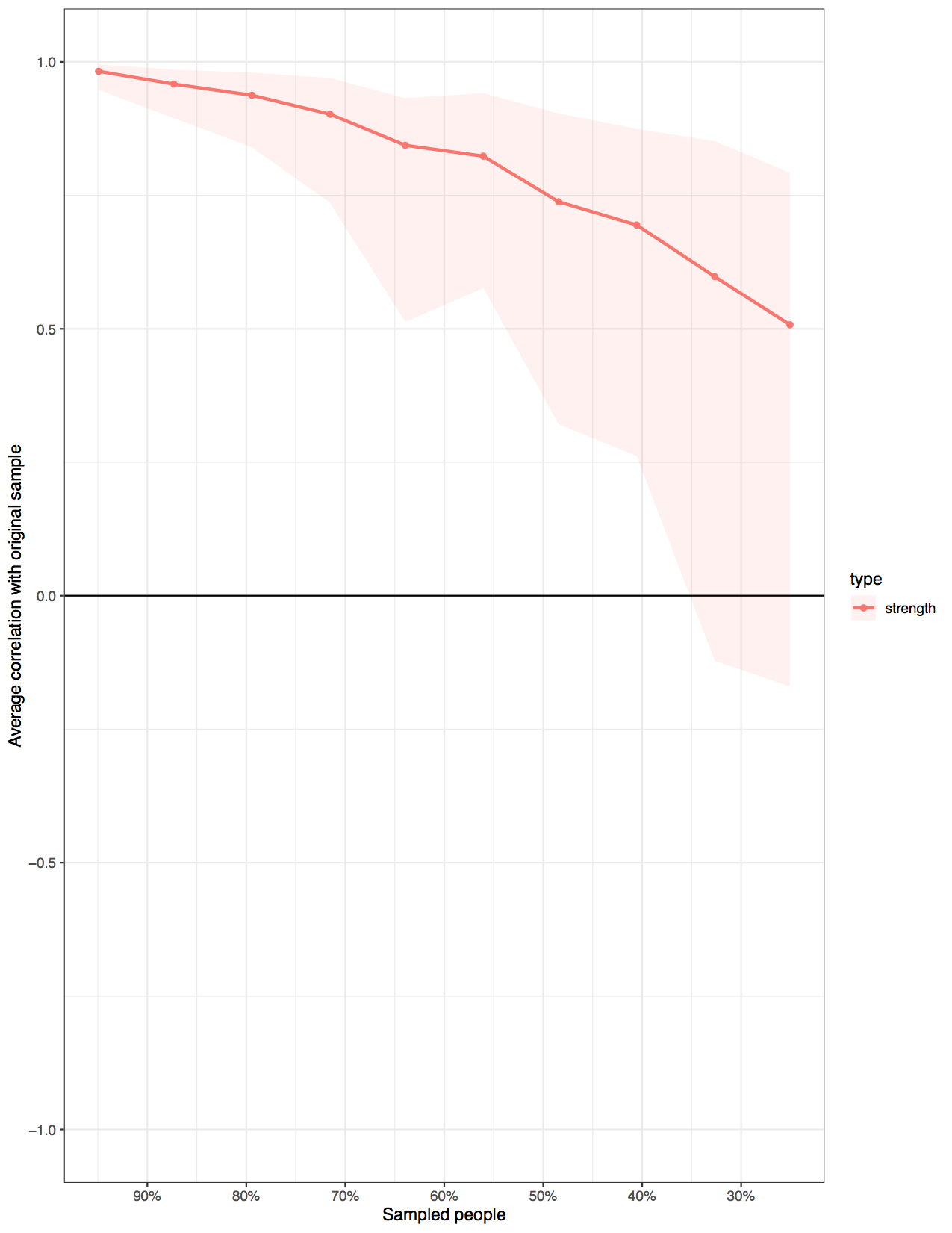


Supplementary Figure 4: Sensitivity analysis of network of CAPS-5 PTSD symptoms with PHQ-15 physical health symptoms using imputed data sample.


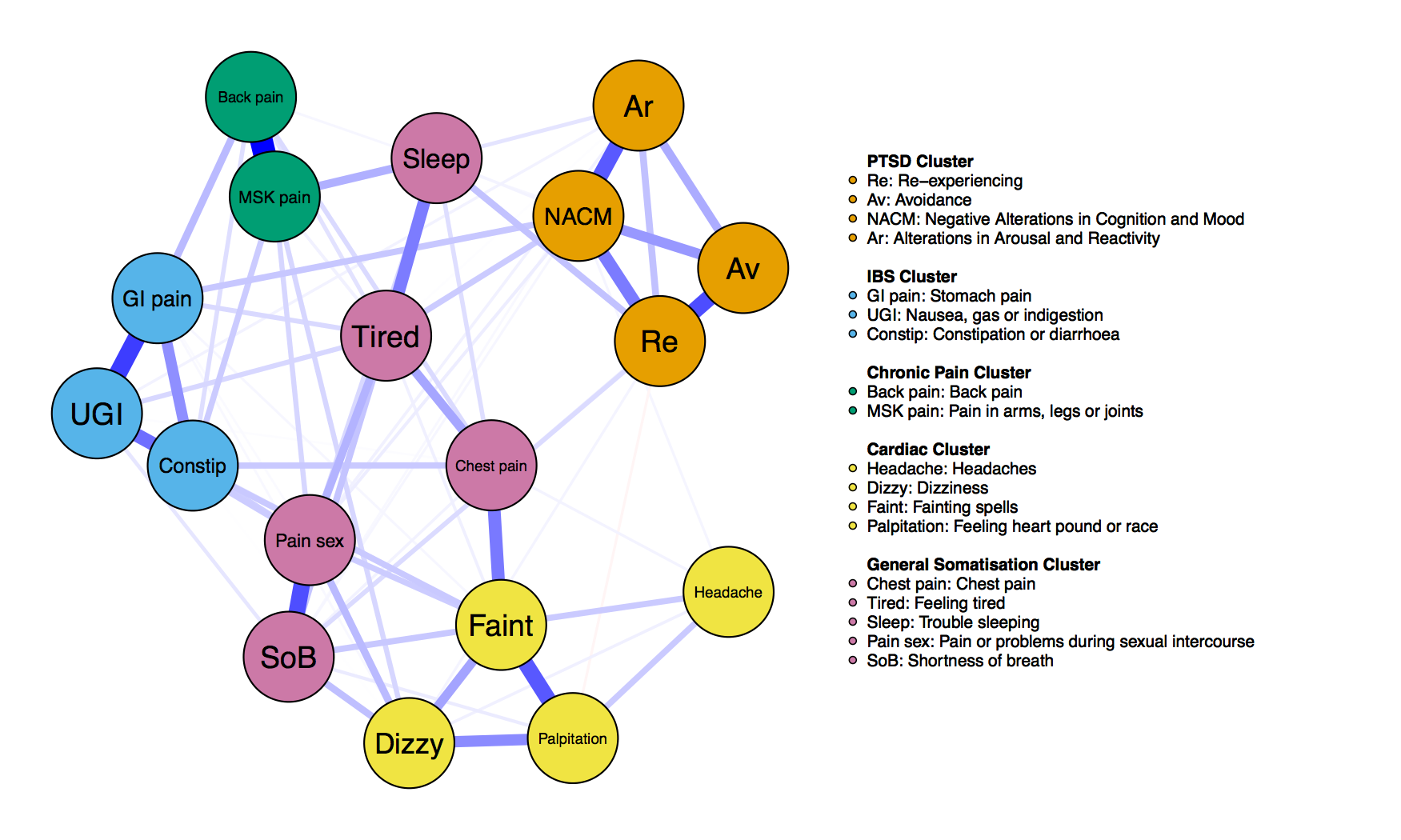

Supplement: Supplementary file 1 — Supporting Material [file JTS-34-375-s002.docx]
