# Supplementary material for: Disturbed Sleep Connects Symptoms of Posttraumatic Stress Disorder and Somatization: A Network Analysis Approach
Source: J Trauma Stress. 2020 Nov 10;34(2):375–83. doi: 10.1002/jts.22619 (PMC9943267; doi:10.1002/jts.22619)
Supplement: Supplementary file 2 — Supporting Material [file JTS-34-375-s003.docx]

| **Supplementary table 1: Participant demographic variables across groups** | | |
| --- | --- | --- |
| **DEMOGRAPHICS** |  |  |
| **Variable** | PTSD (215) | No PTSD (134) |
|  |  |  |
| **Mean age (SD)** | 45.8 (12.03) | 47.5 (13.5) |
|  |  |  |
| **Gender** |  |  |
| **Male (%)** | 99 (46) | 75 (56) |
| **Female (%)** | 116 (54) | 59 (44) |
|  |  |  |
| **Ethnicity (%)** |  |  |
| **White** | 200 (93) | 129 (96) |
| **Non-white** | 15 (7) | 5 (4) |
|  |  |  |
| **Employment** |  |  |
| **Unemployed** | 156 (73) | 71 (53) |
| **Employed** | 58 (27) (data missing for 1 individual) | 63 (47) |
|  |  |  |
| **Living arrangements (%)** |  |  |
| **Married or cohabiting** | 95 (44) | 76 (57) |
| **Single, widowed, divorced or separated** | 118 (56) (data missing for 2 individuals) | 58 (43) |
|  |  |  |
| **Higher education: A levels and above (%)** |  |  |
| **Higher education (achieving at least one A level or above)** | 113 (53) | 87 (65) |
| **No higher education (not achieving at least one A level or above)** | 102 (47) | 47 (35) |
| **PHQ-15 Somatic Symptom Severity** |  |  |
| **High (%)** | 88 (78.6) | 24 (21.4) |
| **Medium (%)** | 31 (49.2) | 32 (50.8) |
| **Low (%)** | 8 (26.7) | 22 (73.3) |
| **Minimal (%)** | 7 (43.8) | 9 (56.3) |
| PTSD = post-traumatic stress disorder, PHQ-15 = Patient Health Questionnaire 15 | | |

| **Supplementary table 2: Frequency of PHQ-15 somatic symptom severity stratified by PTSD diagnosis** | | | |
| --- | --- | --- | --- |
| **PHQ-15 Symptom Severity** | **No PTSD** | **PTSD** | **Chi-square, df, (p-value)** |
| **Minimal** | 9 | 7 | 34.956, 3, (<0.001) |
| **Low** | 22 | 8 |  |
| **Medium** | 32 | 31 |  |
| **High** | 24 | 88 |  |
| PHQ-15 = patient health questionnaire 15 somatic symptom severity scale, PTSD = post-traumatic stress disorder, CPTSD = complex post-traumatic stress disorder | | | |

| **Supplementary Table 3: Frequency of worst trauma by PHQ-15 completers** | |
| --- | --- |
| **Worst trauma as reported on the Life Events Checklist** | **Participant Frequency** |
| **LEC1: Natural disaster** | 1 |
| **LEC2: Fire or explosion** | 7 |
| **LEC3: Transportation accident** | 18 |
| **LEC4: Serious accident at work, home, or during recreational activity** | 6 |
| **LEC5: Exposure to toxic substance** | 0 |
| **LEC6: Physical assault** | 11 |
| **LEC7: Assault with a weapon** | 18 |
| **LEC8: Sexual assault** | 15 |
| **LEC9: Other unwanted or uncomfortable sexual experience** | 36 |
| **LEC10: Combat or exposure to a war-zone** | 13 |
| **LEC11: Captivity** | 1 |
| **LEC12: Life-threatening illness or injury** | 35 |
| **LEC13: Severe human suffering** | 4 |
| **LEC14: Sudden violent death** | 14 |
| **LEC15: Sudden accidental death** | 4 |
| **LEC16: Serious injury, harm, or death you caused to someone else** | 13 |
| **LEC17: Any other very stressful event or experience** | 14 |
| LEC = Life Events Checklist | |

| **Supplementary Table 4: PHQ-15 completers stratified by time since worst trauma** | |
| --- | --- |
| **Time since worst trauma** | **Participant Frequency** |
| **<1 Month** | 28 |
| **1-6 Months** | 83 |
| **6-12 Months** | 42 |
| **1-2 Years** | 53 |
| **2-5 Years** | 2 |
