# Supplementary material for: Disturbed Sleep Connects Symptoms of Posttraumatic Stress Disorder and Somatization: A Network Analysis Approach
Source: J Trauma Stress. 2020 Nov 10;34(2):375–83. doi: 10.1002/jts.22619 (PMC9943267; doi:10.1002/jts.22619)
Supplement: Supplementary file 3 — Supporting Material [file JTS-34-375-s001.docx]

> mat1 <- cor_auto(CAPStest6)

> qgraph(mat1, graph = "glasso", sampleSize = 349 ,layout="spring", legend=FALSE)

> gr1 <- list("PTSD Cluster"=c(1, 2, 3, 4),"IBS Cluster"=c(5, 16, 15), "Chronic Pain Cluster"=c(6, 7), “Cardiac Cluster” = c(8, 10,11,12), ”General Somatisation Cluster" =c(9, 17, 18, 14, 13))

> longnames <- c("Re-experiencing", "Avoidance", "Negative Alterations in Cognition and Mood", "Alterations in Arousal and Reactivity", "Stomach pain", "Back pain", "Pain in arms, legs or joints", "Headaches", "Chest pain", "Dizziness", "Fainting spells", "Feeling heart pound or race", "Shortness of breath", "Pain or problems during sexual intercourse", "Constipation or diarrhoea", "Nausea, gas or indigestion", "Feeling tired", "Trouble sleeping")

> shortnames <- c("Re", "Av", "NACM", "Ar", "GI pain", "Back pain", "MSK pain", "Headache", "Chest pain", "Dizzy", "Faint", "Palpitation", "SoB", "Pain sex", "Constip", "UGI", "Tired", "Sleep")

> net1 <- qgraph (mat1, graph = "glasso", sampleSize = 349, layout = "spring", legend=TRUE , groups = gr1, labels = shortnames , nodeNames=longnames , legend.cex=0.4 , GLratio = 1.5 , theme = "colorblind", posCol = "blue", negCol = "red", filetype = "pdf")

> centralityPlot(net1, scale = "z-scores",include =c("Strength","ExpectedInfluence"), orderBy = "Strength", labels = shortnames)

> cent1 <- centrality_auto(net1)

> b1 <- bootnet(CAPStest6, nBoots=1000, nCores=8, default = "EBICglasso")

> pdf(“EdgeWeightCIs.pdf")

> plot(b1, labels = FALSE, order = 'sample')

> dev.off()

> pdf("EdgeWeightsDiffTest.pdf")

> plot(b1, 'edge', plot = 'difference', onlyNonZero = TRUE, order = 'sample')

> b2 <- bootnet(CAPStest6, nBoots=1000, nCores=8, type='case', default = "EBICglasso")

> plot(b2)

> corStability(b2)
> plot(b1, 'strength', order='sample', labels=TRUE)

> ega1 <- EGA(CAPStest6, plot.EGA = TRUE)

> egagrp <- ega1$wc

> bridge1 <- bridge(net1, communities = egagrp)

> pdf("bridge1.pdf", paper = "a4r", width = 12, height = 6)

> plot(bridge1)

> dev.off()

> b_strength <- bridge1$`Bridge Strength`

> b_strength <- scale(b_strength)

> goldbricker(CAPStest6, p = 0.05, method = "hittner2003", threshold = 0.25,corMin = 0.5, progressbar = TRUE)
